# Evaluation of the Implementation of a Well-being Promotion Program for College Students

**DOI:** 10.3389/fpsyt.2021.610931

**Published:** 2021-02-12

**Authors:** Robyn Long, Megan Kennedy, Katie Malloy Spink, Liliana J. Lengua

**Affiliations:** ^1^Department Psychology, The Center for Child & Family Well-Being, University of Washington, Seattle, WA, United States; ^2^The Resilience Lab, Undergraduate Academic Affairs, University of Washington, Seattle, WA, United States

**Keywords:** resilience, mindfulness, emotion regulation, coping, self-compassion, mental health, college students

## Abstract

Stress that undergraduate students experience is a growing public health concern, and there is increasing attention to programs that promote protective factors and skills to support resilience and well-being. *Be REAL (REsilient Attitudes and Living)* is a program that has been shown to increase students' use of effective coping strategies, mindfulness, and sense of well-being. This study examined whether the program would be effective when delivered by university staff who mentor or advise students.

**Methods:** Eleven university staff advising or mentoring students delivered *Be REAL* in a variety of campus settings to 271 students, and 116 students completed pre- and post-test assessments to evaluate potential changes in stress reduction, managing emotions, coping, social connections, well-being and mental health.

**Results:** Students who participated in Be REAL showed significant pre to post-test improvements in perceived stress, emotion dysregulation, coping, social connection, self-compassion, and symptoms of anxiety. There was also a trend toward improvements in symptoms of depression.

**Conclusions:** The findings suggest that training university staff who work with students to deliver well-being programs is a potential avenue for supporting college student mental health, and a more rigorous evaluation of the *Be REAL* program is warranted.

## Introduction

### College Student Mental Health

Recent years have revealed an upward trend in mental health concerns among college students and young adults. The American College Health Association (1) reports that approximately 40% of undergraduates have felt severely depressed in the last year, and more than 10% have seriously considered suicide (1). Research by the U.S. Department of Health and Human Services shows that one in every four young adults, ages 18–25, has a diagnosable mental health illness (2). Although further study is needed to better understand this phenomenon and its causal factors, there appears to be sufficient evidence to conclude that reported mental health needs of college students are greater than years past and continuing to grow (3–5). The student body at the University of Washington (UW) in Seattle is no exception to these findings. In 2020, the UW participated in the Healthy Minds Study, an annual survey that, among other things, measures rates of student mental health concerns. The results demonstrated that the mental health profile of UW students mirrors the national trends.

The increasing mental health needs of college and university students have become a formidable challenge for higher education institutions that typically have limited resources to expand mental health support (6). The vast majority of university-based counseling center directors report a demand for services that exceed their capacity (7). Mental health advocates frequently call for increased campus mental health staff, however, this may not be a feasible remedy. The Center for Collegiate Mental Health reports that utilization of campus mental health services has increased by 30–40% while institutional enrollment has grown by 5% (8). This finding suggests that even with staffing increases, the exponential growth in need for services is likely to outpace capacity expansion.

The stakes in this dilemma are significant: numerous studies have pointed to a relationship between mental health and academic achievement (9–11). Mental health symptoms are a predictor of a lower GPA and dropping out of college (12). This relation is particularly important when considering the needs of students of color, who are less likely than white students to access and obtain support from campus mental health services (13). Consequently, finding more effective strategies to support student mental health has become a high priority for multiple stakeholders, including university administrators, student affairs professionals, staff counselors, faculty, and students themselves (14–16).

Enlisting the entire campus in promoting and supporting student mental health is advised by leading experts. One of them is the Jed Foundation, a national non-profit organization that advises colleges and universities on improving student mental health and reducing suicide. The foundation's approach is drawn primarily from the overall strategic direction of the United States Air Force (USAF) Suicide Prevention Program, a population-based strategy to reduce risk factors and enhance protective factors for suicide (17). A multilayered approach to student mental health is increasingly gaining attention from campus leadership across the U.S. A recent qualitative and quantitative evaluation, led by Chang et al. (18) of a large public university's current system of care analyzed responses from students, staff, and faculty regarding challenges accessing care, the impact of lacking community and sense of belonging, and the relationship between mental health and academic pressures. The findings concluded that successfully addressing the matter would require a multi-pronged effort and proposed an approach in which the whole campus community participates in creating a culture of well-being (18).

### Interventions to Support College Student Mental Health

When referring to mental health, we are referring both to problems, including anxiety and depression, as well as well-being, including a sense of happiness, satisfaction with life and relationships, and flourishing. In the traditional campus mental health service delivery model, interventions to address symptoms and improve coping skills are offered to students who present to the appropriate office for help. At the UW, trained mental health clinicians offer various group interventions and workshops designed to improve coping with anxiety, depression, relationship problems, and academic stressors. While these topics are relevant for a broad range of students, obtaining the content requires that students feel comfortable accessing mental health services within a counseling center space and sometimes involve a service fee. In addition, with limited service capacity, most services are available only to student presenting with problems. Although most of these types of mental health supports are located within a clinical setting, an attempt to reach a more general student audience might take a number of forms such as credit bearing courses that teach wellness skills (19), group-based programs offered in health settings, web or app based tools (20), and more. In addition, campuses increasingly are taking a preventive or promotive approach to mental health, not only aiming to reduce mental health problems, but also to promote well-being, including resilience, flourishing and happiness.

### The Current Study

The goal of this study was to expand evidence for the effectiveness of a mindfulness-based coping-enhancement program, *Be REAL (Resilient Attitudes & Living)*, when delivered by staff who advise or mentor students at the University of Washington. A previous waitlist control study evaluating the program found that students living in residential halls demonstrated significant improvements in mindfulness, executive control, active coping, self-compassion, social connectedness, resilience, and flourishing. Further, the majority of these changes were maintained at a three-month follow-up and the program demonstrated high student satisfaction and attendance (21).

In this current study, we sought to expand Be REAL into an even more accessible format where students could develop stress-coping skills in the living, learning, and community spaces that they frequent as part of routine college life (e.g., residence halls, academic departments, cultural centers, and affinity group spaces). By offering groups within campus-based services, Be REAL reduces barriers to accessing services, which is particularly crucial for students of color and other marginalized populations less likely to seek out services otherwise (13). Furthermore, Be REAL is designed to be delivered by professional staff who have received training in the content, but they need not have clinical training. This allows for a broad range of campus professionals in diverse roles to model and teach resilience-skills as part of their work with students. Thus, students encounter content that centers well-being within multiple domains of their college experience, and the campus distributes the responsibility for student wellness across units. Finally, we expanded the well-being measures from the prior study to include anxiety and depression in order to assess the program's impact on student mental health. Our specific research questions to evaluate the expansion of Be REAL into a more accessible format included:

1. To what extent does Be REAL hold promise for improving student well-being and mental health, including symptoms of depression and anxiety, when offered by university staff who advise or mentor students?
2. Can Be REAL be feasibly implemented by university staff who advise or mentor students in a variety of campus settings such as a course for credit or in student affinity group spaces?
3. How satisfied will students be with Be REAL when it is delivered by university staff members?

## Methods

### Intervention

*Be REAL (Resilient Attitudes & Living)* includes a mix of contemplative practices (e.g., breathing practices, Hatha yoga sequences, guided meditation) and training in cognitive-behavioral coping and emotion regulation skills (e.g., radical acceptance, balanced decision making, and cognitive reframing). During the 6-week program, students meet once a week for 90 min. Each session highlights skills related to four areas: reducing stress, managing emotions, coping with challenging situations, and building connections and compassion [see Table 1 for an overview; for a detailed description see (21)].

**Table 1:** Summary of content and skills in Be REAL's six sessions.

	**Key topics and practices**
1	Topics: Group introductions, overview of concepts, introduction to the stress response Practices: Tuning into the breath; yoga; mindful listening
2	Topics: Understanding thought patterns, wise mind Practices: Stress check; labeling thoughts; be in the pause breathing; connecting with my values; mindfulness of others
3	Topics: Emotion regulation, coping skills Practices: +2 breathing; yoga; mindfulness of the senses; name it to tame it; holding a stone; 3-2-1 (3 things you can see, 2 things you can touch, 1 thing you can hear); willing hands
4	Topics: Window of tolerance, radical acceptance, common humanity Practices: Tuning into the breath; peace & kindness meditation, progressive muscle relaxation; the 3Ps: pause, be present, proceed; just like me
5	Topics: Cognitive reframing, radical acceptance, self-compassion Practices: +2 Breathing; gratitude meditation; taking in the good; anchor phrases
6	Topics: Interactive review, writing a letter to your future self Practices: Stress check; be in the pause breathing; peace & kindness meditation

### Procedures

In previous research, Be REAL groups were facilitated by professionals with substantial mindfulness training (21). However, the developers intentionally designed the program so that it could be facilitated by staff across a range of roles in higher education (i.e., regardless of their familiarity with mindfulness or clinical training). The Be REAL training model emphasizes competencies in four areas for facilitators:

- *Mindfulness and Self-Compassion Practices*: A foundational awareness of the purpose behind selected contemplative practices and an ability to guide brief exercises (e.g., between 5 and 15 min).
- *Cognitive Behavioral Skills*: Familiarity with psychoeducational topics and skills, such as emotion regulation, radical acceptance, and cognitive reframing.
- *Group Facilitation*: Competence in promoting positive group interactions, normalizing and validating participants' experiences, and facilitating reflection on the practices and skills.
- *Inclusive, Trauma-Informed Teaching*: Skillfulness in creating a supportive and welcoming environment as well as critical self-reflection by facilitators.

The primary Be REAL training model includes staff participation in a 6-week version of Be REAL to experience the program followed by a facilitation training. After staff participate in the 6-week program, they receive the 180-page manual and participate in a 6-h facilitator training. The manual includes scripts for all activities and content, prompts for engaging students in discussions, and resource sections to outline aligning sessions and instruction with trauma-informed mindfulness practices. The training covers how to lead brief mindfulness practices, present cognitive behavioral skills, facilitate group discussions, key points in trauma informed teaching [e.g., (22, 23)] and cultural humility as a framework for critical self-reflection in creating an inclusive learning environment [e.g., (24)]. Recognizing that some university staff may already have expertise in content within Be REAL (e.g., mental health providers), we also developed an abbreviated training that introduces the concepts in Be REAL without the need to complete the six-week program.

As part of training staff on campus to deliver Be REAL, we held introductory meetings with a range of campus partners including mental health centers, units supporting students from underrepresented minority backgrounds, and the recreation department. Staff working in these units are closely familiar with the student communities they support and expressed an interest in being trained to offer Be REAL to students. We then held a 6-week Be REAL program for 12 staff members, six who went on to complete the additional facilitator training. We trained five additional staff members through the abbreviated training model because they were already certified mindfulness instructors or mental health clinicians. A total of 11 staff members were trained to deliver Be REAL.

Staff who had completed either training model volunteered to teach Be REAL to students through their campus units, such as a course for credit, seminar, or affinity group. Staff recruited students through their own program or department channels. The students who enrolled in the groups were then invited to participate in this study by completing online surveys regarding their well-being. A research coordinator emailed students with a link to sign up and the staff facilitators shared study details with participants. Enrollment in the study was voluntary and staff were not informed which students participated in the research. When students enrolled in the study, they provided informed consent and completed one survey a week before their Be REAL group (pre-test) and one survey 1 week after their group ended (post-test). Study data were collected and managed using REDCap electronic data capture tools hosted at the University of Washington (25). REDCap (Research Electronic Data Capture) is a secure, web-based software platform designed to support data capture for research studies. Students were compensated $10.00 and $15.00 for completing the pre- and post-test surveys, respectively. Students were also asked to complete a feedback survey at the end of their group's final session. Groups were offered in academic quarters starting winter 2019 through spring 2020.

### Recruitment

**Figure 1:**
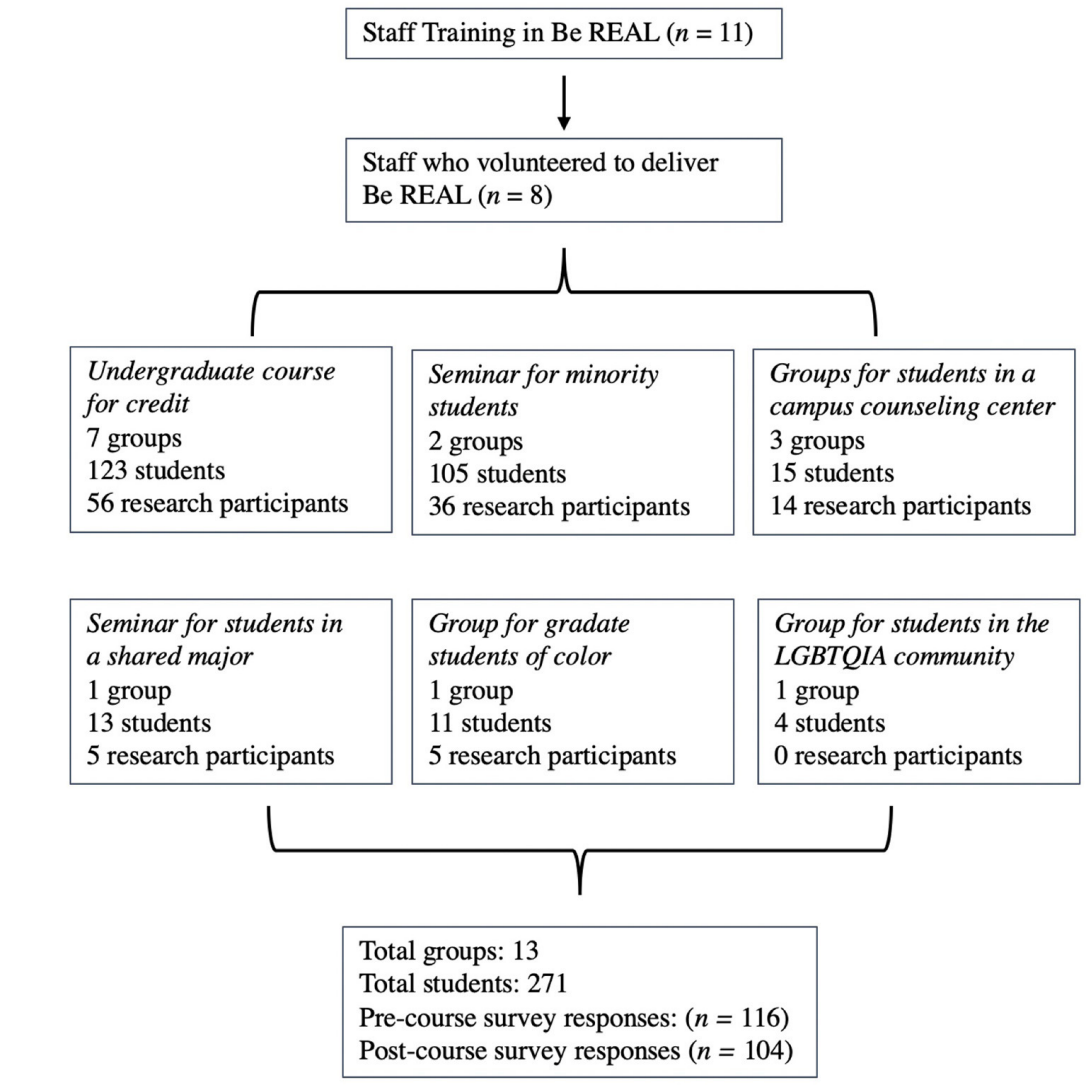
Study implementation.

Over a 14-month period, eight staff members volunteered to facilitate a total of 15 groups in six different settings (Figure 1), including units that served students from historically marginalized communities, which included:

- An undergraduate course for credit (7 groups, 123 students total), facilitated by a program manager from the campus recreation department.
- A seminar for undergraduate students from an underrepresented community engaged in a cohort-based program (2 groups, 105 students total), many who identify as students of color, co-facilitated by two advisers with similar racial, ethnic and/or linguistic background as the students.
- A group for students on a waitlist to receive mental health services at one of the campus-based counseling centers (3 groups, 15 students total), facilitated by mental health clinicians.
- A seminar for undergraduate students in a shared major (1 group, 13 students), facilitated by an academic advisor.
- A group for graduate students of color (1 group, 11 students), co-facilitated by two advisors who identified as people of color.
- A group for students with gender- and sexual-diverse identities and expressions (1 group, 4 students), facilitated by an advisor who is also part of the LGBTQIA community.

As shown in Figure 1, a total of 271 students enrolled in the 15 Be REAL groups and 43% (*n* = 116) volunteered to participated in research and completed the pre-test assessment. Of the research participants, 88 students (76%) completed the post-test assessment. Eligibility included being an undergraduate student, at least 18 years old, and able to read, write, and speak English.

The vast majority of research participants were undergraduate students (96%) while a small percentage were graduate students (4%). Students identified as 79% women, 18% men, 1% gender fluid, 1% gender non-conforming, and 1% Other. Participants included 40% Latinx or Hispanic, 34% Asian, 20% White, 3% African American/Black, and 3% Other. Nine percent of the participants identified as international students. Two-thirds of the participants (66%) reported receiving financial aid. A majority of the students reported their parents did not have a college degree (parent 1, 54%; parent 2, 57%), with 44% indicating neither parent had a college degree. Twenty-five percent reported receiving other mental health support services.

Compared to the general U.S. college population, the sample of Be REAL participants is overrepresented by women and underrepresented by men. This phenomenon is reflective of the fact that on college campuses, women generally tend to seek mental health programming at higher rates than men. However, the second-largest faction of participants (*n* = 105) was part of a seminar for undergraduate students from an underrepresented community–of which a significant majority of women enrolled. Similarly, the undergraduate course for credit generally had more women enrolled than men.

### Measures

At both time-points, participants completed self-report measures assessing mindfulness, perceived stress, emotion regulation, executive control, coping, social connectedness, self-compassion, anxiety, depression, and indicators of well-being including resilience, flourishing, and happiness. Measures were selected to assess the targets of the program including reducing stress, improving emotion regulation, enhancing active coping, and building connections, as well as the expected impact of the program on well-being and mental health problems.

**Dispositional mindfulness** was assessed using the 15-item Mindful Attention Awareness Scale (26) which assesses present attention or lack of awareness. Participants rate statements such as “I find myself doing things without paying attention,” and “I rush through activities without being really attentive to them,” on a 6-point scale (1 = almost always−6 = almost never). Internal consistency of 0.80–0.87 has been reported, and alpha was 0.88 in this study.

**Perceived stress** was assessed using stressfulness ratings on the General Life Events Schedule (27) which includes 18 moderately and highly stressful life events, such as moving, losing a job or friend. Respondents indicated whether each of events occurred in the past year and, if it occurred, how stressful it was on a 3-point scale (not stressful, a little stressful, very stressful). Life event examples included “You moved or there was a change in your living situation,” and “A close family member had medical problems.” Scale scores were the sum of the stressfulness ratings. Cronbach's alpha is inappropriate for life events scales because the occurrence of these life events is assumed to be independent. This measure has been used broadly in the literature (28) and been shown to predict adjustment problems and substance use in adolescents (29).

**Emotion Dysregulation** was measured using the brief 18-item Difficulties in Emotion Regulation Scale (30), scored so that it represented deficits in awareness, understanding, and acceptance of emotions, impulse control, and access to emotion regulation strategies. Participants rate statements such as “I pay attention to how I feel,” and “When I am upset, I become out of control,” on a 5-point scale (1 = almost never−5 = almost always). Internal consistency reliability of 0.97 has been reported, and was 0.79 in this study.

**Executive control** was assessed using the attention (5 items) and inhibitory control (7 items) subscales of the Adult Temperament Questionnaire–Short Form (31). Participants rate statements such as “I am often late for appointments,” and “I can keep performing a task even when I would rather not do it,” on a 7-point scale (1 = extremely untrue−7 = extremely true). Attention control reflects the capacity to focus and shift attention to relevant stimuli, and inhibitory control assesses the capacity to suppress inappropriate approach behaviors. Internal consistency of the combined subscales in this study, measured by Cronbach's alpha, was 0.76.

**Coping** was assessed using the short form of the COPE inventory (32), which asks participants what they do or feel during a stressful event We used 24 items assessing 8 types of coping behaviors (3 items each), with response options ranging from 1 = “I usually don't do this at all” to 4 = “I usually do this a lot.” Disengagement strategies include *denial* (i.e., “I refuse to believe that it has happened”) and *distraction* (i.e., “I turn to work or other substitute activities to take my mind off things”). Engagement strategies include *active* (i.e., “I concentrate my efforts on doing something about it”), *planning* (i.e., “I make a plan of action”), *restraint* (“I force myself to wait for the right time to do something”), *positive reappraisal* (i.e., “I try to see it in a different light, to make it seem more positive”), *humor* (i.e., “I laugh about the situation”), and *acceptance* (i.e., “I get used to the idea that it happened”). Alphas for the subscales were: active = 0.70, planning = 0.71, positive reappraisal = 0.69, acceptance = 0.66, denial = 0.71, and disengagement = 0.60.

**Self-Compassion** was measured using the 12-item Self-Compassion Scale-Short Form (33) which assesses dimensions of self-kindness, self-judgement, common humanity, isolation, mindfulness, and over-identification on 5-point scale (1 = almost never−5 = almost always). Response options include “I'm intolerant and impatient toward those aspects of my personality I don't like,” and “I try to see my failings as a part of the human condition.” Internal consistency of 0.80–0.92 has been reported, and was 0.87 in this study.

**Social connection** was assessed with the 14-item Positive Relations with Others subscale of the Psychological Well-being measure (34). The positive relations measure assesses the extent to which an individual has satisfying relationships with others, concern for others, is capable of empathy, and understands give and take of relationships. Participants respond with “yes” or “no” to statements such as “Most people see me as loving and affectionate,” and “I don't have many people who want to listen when I need to talk.” Ryff (34) reported a test-retest reliability of 0.83, internal consistency reliability of 0.91, and indicated validity by associations with higher life satisfaction and self-esteem. Alpha in the present study was 0.82.

**Well-being** was indicated with measures of resilience, flourishing, and happiness. On the 6-item Brief Resilience Scale (35) respondents indicated on a 5-point scale their ability to cope with and recover from stressful situations (1 = strongly disagree−5 = strongly agree). Examples include “I tend to bounce back quickly after hard times,” and “I have a hard time making it through stressful events.” Internal consistency ranging from 0.80 to−0.91 has been reported, and was 0.87 in this study. Well-being was also assessed using the 8-item Flourishing Scale (36). Respondents indicate on a 7-point scale (1 = strongly disagree−7 = strongly agree) their agreement with items such as “I am engaged and interested in my daily activities,” and “I lead a purposeful and meaningful life.” Alpha was 0.89. Happiness was measured with the Subjective Happiness Scale (37), a 4-item measure assessing trait happiness. Each item uses a Likert scale from 1 to 7, and an example item is “In general, I consider myself:” with response options of 1=Not a very happy person to 7=A very happy person. The internal consistency of this measure is between 0.79 and 0.94 across a range of samples (37) and 0.85 in this study.

**Mental health** was assessed as symptoms of anxiety and depression. Anxiety symptoms were measured using the widely-used 7-item Generalized Anxiety Disorder (GAD-7) scale designed to briefly assess probable generalized anxiety disorder (38). Participants rate statements such as “Feeling nervous, anxious or on edge,” and “Worrying too much about different things,” on a 4-point scale (0 = not at all−3 = nearly every day). Adequate reliability, construct and criterions related validity have been reported (38). Internal consistency was 0.88 in this study. Depression symptoms were assessed using the 9-item Public Health Questionnaire (PHQ-9), which is a brief measure of depressive symptom severity (39). Participants rate statements such as “Little interest or pleasure in doing things,” and “Feeling down, depressed, or hopeless,” on a 4-point scale (0 = not at all−3 = nearly every day). Internal consistency has been reported to be 0.86–0.89, and was 0.85 in this study.

**Analysis Plan:** We used paired or dependent samples *t*-tests to test mean differences between pre-test and post-test on each outcome. Intent-to-treat analyses were used in which missing post-test values were substituted with available pre-test values. As a result, all analyses were conducted on the sample of 116 participants who completed the pre-test assessments. This addresses bias that might be introduced by attrition and retains power for analyses, and the pattern of significant findings were identical to those when unsubstituted values were used (40, 41). Prior to conducting these tests, correlations of pre-test measures with potential covariates were examined. Variables that were significantly correlated with pretest levels of study measures were examined as potential moderators of pre- to post-test changes using general linear modeling. Intervention effect sizes were estimated using the repeated-measures *d* (42). Benjamini–Hochberg correction for false discovery rate (43) was used to address alpha inflation given multiple comparisons. Reported *p*-values are unadjusted, with those remaining significant after correction in bold text.

## Results

### Preliminary Analyses

Distributional properties of the variables were examined. Variable ranges indicated plausible values and reasonable variability for all variables. Measures of skewness (|0.04|–|1.18|, M = 0.30) and kurtosis (|0.02|–|1.45|, M = 0.51) were acceptable, indicating that assumptions of normality were not violated.

Correlations among pre-test measures and potential covariates are presented in Table 2. Potential covariates examined included participant sex, neither parent having a college degree, currently receiving other mental health or substance use treatment, international student, receiving financial aid, the term the student participated in the program (i.e., higher value is later in the year), and whether the program was delivered in-person or online (online classes were made available only in one quarter as a result of COVID19 social distancing orders). We also examined the number of classes the student participated in by the end of the program to determine whether participant characteristics were related to attendance. We did not consider student level (undergraduate or graduate) since the vast majority were undergraduates (96%).

**Table 2:** Correlations of study variables with potential covariates.

	**Sex**	**First college**	**Other mental health**	**Internal**	**Financial aid**	**Term**	**Online vs. in-person**	**Number of classes**
**REDUCING STRESS**								
Mindfulness	0.14	0.07	−0.23^*^	−0.05	0.08	0.09	−0.00	0.19
Perceived stress	0.05	0.16	0.21^*^	−0.13	0.27^*^	0.09	0.04	−0.08
**MANAGING EMOTIONS**								
Emo dysregulation	0.07	−0.10	0.10	0.12	−0.09	0.02	0.20^*^	−0.17
Executive control	0.09	0.17	−0.26^*^	−0.11	0.09	−0.06	0.03	0.07
**COPING WITH CHALLENGING SITUATIONS**								
Active	0.02	0.07	−0.05	−0.10	0.21^*^	0.03	0.05	−0.02
Planning	0.11	0.14	−0.09	−0.11	−0.06	−0.06	0.01	−0.03
Reframing	−0.03	0.10	−0.10	−0.08	0.12	−0.00	0.15	−0.04
Acceptance	−0.01	−0.01	−0.17	−0.12	−0.06	−0.06	0.09	−0.03
Denial	0.11	0.10	−0.04	0.12	0.07	0.02	−0.04	−0.25^*^
Disengagement	0.09	0.17	0.11	−0.13	0.20^*^	0.03	0.08	0.05
**BUILDING CONNECTIONS AND COMPASSION**								
Social Connection	0.01	−0.05	−0.07	−0.05	−0.01	0.09	0.01	0.26^*^
Self-compassion	−0.08	−0.07	−0.17	−0.09	−0.04	0.04	0.02	0.08
**WELL-BEING**								
Flourishing	−0.05	−0.00	0.17	0.08	−0.03	0.07	0.04	−0.20^*^
Resilience	−0.07	−0.00	−0.11	−0.18	0.07	0.09	0.08	0.05
Happiness	0.09	−0.08	−0.21^*^	−0.12	0.09	0.09	0.02	0.07
**MENTAL HEALTH PROBLEMS**								
Anxiety	0.08	0.12	0.22^*^	0.03	−0.04	−0.05	0.02	−0.15
Depression	0.01	0.14	0.23^*^	0.14	0.01	0.03	0.12	−0.18

*Sex is coded Male = 0, Female = 1*.

* *p < 0.05*.

Participants receiving other mental health or substance use services reported lower mindfulness, happiness, and executive control, and higher perceive stress, anxiety, and depression symptoms. Students receiving financial aid reported higher perceived stress, more active and disengagement coping. Participants who engaged in higher in denial, were less socially connected, and were higher in flourishing attended fewer program sessions. Receiving the program online was related only with higher emotion dysregulation. This could be related to the start of COVID19 which necessitated that the program be delivered online. However, given that there were no other variables related to online delivery, this was not included as a covariate. None of the other variables were correlated with pre-test study measures. Receiving other mental health services, financial aid and number of sessions attended were examined as potential moderators in subsequent analyses.

### Test of Program Effects

In order to assess our first aim, to explore if Be REAL holds promise for improving student well-being and mental health when offered by university staff, we analyzed students pre-post survey results (Table 3). Two variables assessed the program's impact on reducing stress: mindfulness and perceived stress. There was no significant difference from pre- to post-test on mindfulness. However, the number of sessions attended moderated the effect, such that there was a greater increase in mindfulness for participants who attended more sessions [F_(1, 112)_ = 8.64, *p* = 0.004]. There was a significant decrease in participants' reports of perceived stress that was not moderated by covariates.

**Table 3:** Tests of mean differences from pre-treatment to post-treatment using intent-to-treat analyses (*n* = 116).

	**Pre-test M(SD)**	**Post-test M(SD)**	***t-*test (*df* = 115)**	***p***	**Pre-/Post-test *d_***repeatedmeasures***_***
**REDUCING STRESS**					
Mindfulness	3.44 (0.79)	3.43 (0.75)	0.19	0.853	0.024
Perceived stress	**13.69 (7.35)**	**11.75 (7.31)**	**4.06**	**0.000**	**0.455**
**MANAGING EMOTIONS**					
Emo dysregulation	**1.77 (0.57)**	**1.64 (0.61)**	**2.97**	**0.004**	**0.305**
Executive control	3.12 (0.75)	3.13 (0.55)	0.11	0.915	0.000
**COPING WITH CHALLENGING SITUATIONS**					
Active	**1.66 (0.69)**	**1.81 (0.71)**	**2.38**	**0.019**	**0.262**
Planning	**1.66 (0.65)**	**1.87 (0.71)**	**3.14**	**0.002**	**0.338**
Reframing	**1.46 (0.92)**	**1.76 (0.72)**	**3.42**	**0.001**	**0.364**
Acceptance	**0.67 (0.72)**	**1.80 (0.70)**	**9.75**	**0.000**	**1.031**
Denial	**0.55 (0.62)**	**1.37 (0.85)**	**-9.79**	**0.000**	**1.195**
Disengagement	**1.58 (0.71)**	**1.85 (0.78)**	**-3.22**	**0.002**	**0.345**
**BUILDING CONNECTIONS AND COMPASSION**					
Social connection	**0.44 (0.21)**	**0.69 (0.22)**	**8.60**	**0.000**	**1.017**
Self-compassion	**1.67 (0.69)**	**2.07 (0.40)**	**6.20**	**0.000**	**0.648**
**WELL-BEING**					
Flourishing	1.63 (0.93)	1.63 (0.75)	0.03	0.980	0.000
Resilience	2.95 (0.76)	2.84 (0.73)	1.01	0.314	0.103
Happiness	3.14 (1.30)	3.20 (1.11)	0.66	0.513	0.072
**MENTAL HEALTH PROBLEMS**					
Anxiety	**9.28 (5.50)**	**7.69 (4.98)**	**3.85**	**0.000**	**0.412**
Depression	9.32 (5.48)	8.47 (5.64)	1.79	0.077	0.160

*Bolded values indicate tests that remain statistically significant at the p < 0.05 level after Benjamini–Hochberg correction. Reported p-values above are unadjusted*.

Two variables were examined to assess the program's impact on managing emotions: emotion dysregulation and executive control. There was a significant decrease in emotion dysregulation, but no change in self-reported executive control. Neither effect was moderated by number of sessions attended or other mental health services.

To assess the program impact on coping, we examined the effects on 6 specific coping dimensions representing engagement and disengagement coping. There were increases in all engagement coping dimensions, active, planning reframing and acceptance. Increases in active coping were moderated by number of sessions attended, with greater increases associated with more sessions attended [F_(1, 112)_ = 6.035, *p* = 0.016]. However, there were also increases in disengagement coping, including denial and disengagement, which was in the direction opposite than expected. These effects were not moderated by number of sessions attended or other mental health services.

There was a significant increase in self-compassion that was not moderated by the covariates. There was an increase in social connectedness that was moderated by number of sessions attended, with greater increases in social connectedness for those who attended more session [F_(1, 112)_ = 4.983, *p* = 0.028).

Participants did not demonstrate significant increases on any of our measures of well-being (flourishing, resilience, happiness). However, there was a significant decrease in anxiety symptoms and a trend toward a decrease in depression symptoms. These effects were not moderated by covariates.

### Feasibility and Acceptability

In order to assess our second and third aims, of program feasibility and student satisfaction, we analyzed student attendance and feedback. The Be REAL program exhibited high feasibility and overall acceptability. Of the research participants for whom we have attendance (*n* = 104), 88% attended four or more of the six total sessions. Students (*n* = 184) who completed feedback surveys during the last session also reported high satisfaction and positive feedback. A variety of questions were rated on a Likert scale: Strongly Agree (SA), Agree (A), Neutral (N), Disagree (D), and Strongly Disagree (SD). Examples questions include:

- “The information presented was useful”
- “The practices in class helped me to learn”
- “The program helped me learn skills for managing emotions”
- “The instructor was clear and engaging”
- “I would recommend Be REAL to a friend”

The survey also asked six open-ended questions, such as “Please give examples of the practices you use and how they have changed how you respond to daily life”; “What did you like the most about the program?”; “Do you have any specific feedback for your instructor?.” Examples of student responses include:

- *I never realized how much breathing helped my mental well-being until I ended up missing some of the sessions*.
- *The Be REAL program gave me the tools to regulate my emotions in a healthy way and calm down during stressful events*.
- *The breathing exercises are something that I use often–they help me handle stress such as the* +*2 breathing. I use this a lot while I study*.
- *When I felt like I am getting sidetracked on my homework I would use meditation techniques to clear my mind*.
- *It's the most safe and understanding class I've ever had at [this school]. I feel very confident to learn, be involved and be myself in the class*.
- *The engagement of the instructor. She always felt so open and welcome as well as listened to whatever I brought up. It was so comforting knowing my presence was being acknowledged*.
- *The multiple in-class practices. I was always looking forward to going to class on Tuesday because I knew it would make me feel better*.
- *I liked the fact that we met weekly because it helped form the good habits, and I really like the Be REAL resources site. I keep it bookmarked to go to when I feel stressed*.
- *What I liked the most about the program were strategies to reduce stress and cope with challenging situations*.
- *The different methods we learned was what I liked the most since they are all different and we can have one that best fits us*.
- *It was nice to see that I was not the only person struggling with my mental health and that many people had similar issues*.

## Discussion

This study demonstrated that a student well-being program led by university staff who advise or mentor students is effective in promoting student mental health, emotion dysregulation, self-compassion, social connection, and coping. Our findings also indicate such a program is feasible in various campus environments, such as affinity groups, required seminars, and a course for credit. As outlined earlier, this study also aimed to examine the implementation of Be REAL in this context alongside findings from the previous study of Be REAL with students in residential halls. Below is a discussion of how our findings relate to each aim.

1. *To what extent does Be REAL hold promise for improving student well-being mental health, including symptoms of depression and anxiety, when offered by university staff who advise or mentor students?*

In our prior evaluation of Be REAL we demonstrated improvements in undergraduate students' stress management, emotion regulation, coping and well-being when the program was delivered by facilitators who were part of the research team. In this study, we examined the extent to which Be REAL would continue to have a beneficial impact on students when university staff who support students in a variety of campus settings delivered the program.

In this study, we observed improvements in students' perceived stress, emotion regulation, active and engagement coping strategies, social connectedness, and self-compassion. Additionally, students reported significant decreases in anxiety and a trend toward a decrease in depression. Students reported increases in denial and disengagement coping strategies, which was in the direction opposite expected or intended. However, this finding is consistent with the results of the prior study. It is possible that some items in these coping measures are tapping into students' “radical acceptance” practices; however, more attention is needed in the program to ensure it is not promoting coping strategies that are often correlated with poor mental health. The students also did not report improvements in measures of well-being, which was inconsistent with the prior study. It may be valuable to note that the students in the current study reported higher perceived stress, were more likely to receive financial aid, less likely to have a parent who graduated from college, and more likely to be receiving mental health services. Taken together this sample might have been experiencing more distress than the sample in our prior study, and improvements in mental health were more salient, and might precede future improvements in well-being. Unfortunately, we did not obtain follow-up data to test this hypothesis. Overall, the findings indicate that Be REAL can improve student coping and mental health when delivered by university staff members who advise or mentor students in a variety of settings.

2. *Can Be REAL be feasibly implemented when delivered by university staff who advise or mentor students in a variety of campus settings, such as course for credit or in student affinity spaces?*

The significant effects across target domains and largely replicated effects from our prior study suggest that the implementation model utilized in this study can be a feasible and effective way to deliver well-being programs to college students. Further, the high rates of attendance suggest that the program is feasible in a variety of settings for students. Staff hailed from a variety of campus departments and units serving students, including a number of offices that support students from underrepresented minority groups and students who experience adversity. This highlights a key benefit of this approach for implementation of a program like Be REAL, that is, that barriers to obtaining support might be minimized and accessibility enhanced. Staff training was systematic; however, it did not pose an undue burden on either the staff conducting training or those being trained, also enhancing feasibility. In fact, staff facilitators also reported high satisfaction teaching Be REAL, with 100% of facilitators saying they would recommend learning to facilitate Be REAL to their colleagues. Additionally, all staff members reported that offering the program helped them feel more connected to the students. It is highly encouraging that staff did not report feeling that Be REAL was a burden, but instead that it enhanced their experience and skills. These points are illustrated in the following quotes from staff who facilitated Be REAL:

“*It's [facilitating Be REAL] been great! I love this material and getting to teach it and facilitate the learning of it from such a great curriculum has been fun. Getting feedback from group members that it's helpful is rewarding.”*

“*It was sweet to explore some new tools that I have not practiced before and see the benefit of them. It was also humbling to read student reflections and see what they are going through in their day-to-day lives on campus. These practices work and it was neat to see them utilizing them.”*

In addition, students participating in the study represented a range of diverse backgrounds. They reported high satisfaction with the program and the facilitators offering the program, suggesting that working with campus staff who are already serving students might facilitate students' openness to the program. Students from affinity groups reported that having space for their specific community was supportive. For example, students shared “*I liked that my group was specifically for People of color,” and “Having people of color only was so important and helpful for me.”* Regarding a course for credit, students appeared to have valued the opportunity to receive academic credit for learning skills for well-being:

“It [having Be REAL as a course] was nice to earn a credit while focusing on my mental health.”

“It [having Be REAL as a course] made the practices more of a priority for me since they were for credit.”

“It didn't feel like a class, I looked forward to coming every week. There was no stress of a grade so that we could focus on the practices.”

These findings indicate that such a program is feasible in various campus environments, such as student support services, affinity groups, required seminars, and credit-bearing courses. The Steve Fund and The Jed Foundation—leading experts in young adult mental health—recommend developing tailored interventions for students of color as part of their “Equity in Mental Health Framework” (2017). Included in the framework's set of recommendations is the guidance to offer campus-based programs in varied and culturally relevant formats and to collect data on the effectiveness of these programs. To this end, Be REAL attempts to contribute to the scientific evidence regarding programs and services that seek to support the mental health and well-being of students of color in a university setting. As noted earlier, students of color are less likely than white students to access and obtain support from campus mental health services (13). Thus, offering programs such as Be REAL in affinity spaces and in programs explicitly offered to diverse students holds promise as one avenue through which students of color can access preventative mental health services. Overall the implementation of Be REAL through professional staff holds promise for promoting student mental health and coping.

3. *How satisfied will students be with be real when it is delivered by university staff members?*

In the current study, students reported overall strong satisfaction with the program. Student satisfaction was, however, slightly lower than previous research on Be REAL when led by instructors who were part of the research team. For example, in the prior study, a large majority of students strongly agreed that Be REAL helped them learn skills for managing emotions (71%), whereas fewer students in the current study did (47%). Similarly, students were more confident in the instructor knowledge in the prior study with 90% strongly agreeing that they were knowledgeable, compared to 72% in this study. Of note, only 38% of students in the current study strongly agreed that the program met their goals, compared to 60% in the prior study. This might reflect the fact that in some cases the program was delivered for credit as a course, and in some settings, it was incorporated into a required program of study, as discussed below. These satisfaction ratings point to future directions for implementation of Be REAL in terms of enhancing instructor training and providing more clear guidelines around the context it is more likely to be well-received. Nonetheless, overall, there was high satisfaction with the program, indicating that most students perceived a benefit from participation.

A key contributing factor to these differences in student satisfaction may be that participants in groups with required attendance (e.g., seminars or a course for credit) may have had less intrinsic motivation regarding participation in the program than students who volunteered in previous studies on Be REAL. As a result, the information and skills presented may not have seemed as relevant to them. Additionally, these differences could speak to the variations in training between staff facilitators and certified mindfulness instructors. That is, greater skillfulness and ease in teaching content comes with practice. Evidence from implementation of social-emotional learning (SEL) programs in school settings indicates that the effectiveness of SEL training is a function of both strong curricula and implementation fidelity and skill (44, 45) highlighting the need for adequate facilitator training. An area where the current study was rated higher by students was partner activities. In the current study, 35% of students strongly agreed that these were useful while 19% of students from the original study strongly agreed. After the original study, the developers shared student feedback with staff facilitators who reflected on lower satisfaction with partner activities. In response, they broke up a few of the writing activities and large discussions into small groups or dyads. These changes appear to have improved student satisfaction with partner activities. The overall positive feedback is an indication that the higher education staff are well-positioned to offer skill building programs such as Be REAL.

### Practical and Clinical Implications

College campuses worldwide are grappling with high rates of mental health disorders among students and limited resources. The World Health Organization (WHO) World Mental Health International College Student Initiative has reported that one-third of nearly 14,000 college students across 8 countries screen positive for at least one 12-month mental health disorder (10). Campuses are seeking effective interventions that are financially feasible and reduce barriers to student engagement. This study demonstrated that programs such as Be REAL, which can be embedded into existing programs and settings that are part of students' regular campus experiences, are one potential solution. Our findings are further relevant as interventions involving mindfulness and cognitive behavioral skills gain traction on college campuses in many global settings (46–48).

Furthermore, the recommendation is to adopt a public health approach to social and emotional learning (SEL) in the K-12 system (49). Be REAL is an example of a systemic effort to promote SEL in a coordinated way across college campuses. By providing the programmatic infrastructure and curriculum needed to build university professionals' social-emotional competencies and by promoting the development of social and emotional skills throughout the campus community, Be REAL is taking a strategic approach to the integration of SEL within a higher education context.

Be REAL is not a clinical intervention. However, as a preventive or promotive program, it could address pre-clinical levels of stress and distress in students, or prevent mental health problems from developing, potentially reducing the demand for clinical services through campus counseling centers. It is a model for shifting a campus culture toward greater well-being for students as well as staff and faculty. Be REAL equips staff with well-being skills, that they can in turn impart to students formally through groups and informally through individual advising and mentoring. This parallel process shifts the overall environment for students. For example, widespread well-being programming can increase mental health literacy, normalize discussions about emotions and challenges, and de-stigmatize mental health disorders.

### Limitations and Future Directions

This research built on a previous evaluation of Be REAL by examining the program's implementation in new campus settings when delivered by university staff who work with students, and by assessing its impact on student mental health. Still, there were a number of limitations. First, it was not possible to randomize participants. However, the current design provides an indication of whether students voluntarily signed up for Be REAL through different campus environments and groups. Second, most of the groups did not have enough study participants to analyze their unique group which means we were unable to look at potential differences between student well-being in more depth. Third, this study sample lacked a comparison group, and there was a relatively a low participation in the assessments, resulting in potential bias in the students who participated. Fourth, all measures were self-report, and future research could include clinical or biological measures for stress. Finally, this study did not include a qualitative component, which could allow students to reflect and share more on their experience participating in a well-being program through their specific department, class, or affinity group. This might be particularly important for amplifying the experiences of students who come from communities with oral traditions. All of these areas are critical ones for future research to explore. Additionally, research could examine the effects of Be REAL on staff self-efficacy as program such as Be REAL could potentially strengthen their capacity and tools to address students' and their own stress.

In sum, this study highlights that engaging a broad range of campus professionals in supporting student well-being helps distribute the responsibility for well-being beyond the traditional counseling services. Further, it creates new pathways for students from marginalized communities to receive support who might otherwise experience barriers to care. Be REAL equips university staff advising or mentoring students with cognitive-behavioral skills and mindfulness and compassion-based practices to model, teach, and practice with students. Engaging staff in Be REAL also recognizes the interconnectedness between staff and student well-being; investing in both is necessary for nurturing a healthy campus culture.

## Data Availability Statement

The raw data supporting the conclusions of this article will be made available by the authors, without undue reservation.

## Ethics Statement

The studies involving human participants were reviewed and approved by University of Washington Human Subjects Division. The patients/participants provided their written informed consent to participate in this study.

## Author Contributions

RL and LL designed the study, led the staff trainings, and contributed to the manuscript. MK led campus outreach to recruit staff and students and contributed to the manuscript. LL conducted the majority of the analyses. KM participated in student recruitment, contributed to the analyses, and the manuscript.

## Conflict of Interest

The authors declare that the research was conducted in the absence of any commercial or financial relationships that could be construed as a potential conflict of interest.
